# Feature selectivity is stable in primary visual cortex across a range of spatial frequencies

**DOI:** 10.1038/s41598-018-33633-2

**Published:** 2018-10-16

**Authors:** Brian B. Jeon, Alex D. Swain, Jeffrey T. Good, Steven M. Chase, Sandra J. Kuhlman

**Affiliations:** 1 Center for Neural Basis of Cognition, Carnegie Mellon University, Pittsburgh, USA; 2 Department of Biomedical Engineering, Carnegie Mellon University, Pittsburgh, USA; 3 University of Pittsburgh Integrative Systems Biology Program, Pittsburgh, USA; 4 Department of Biological Sciences, Carnegie Mellon University, Pittsburgh, USA

## Abstract

Reliable perception of environmental signals is a critical first step to generating appropriate responses and actions in awake behaving animals. The extent to which stimulus features are stably represented at the level of individual neurons is not well understood. To address this issue, we investigated the persistence of stimulus response tuning over the course of 1–2 weeks in the primary visual cortex of awake, adult mice. Using 2-photon calcium imaging, we directly compared tuning stability to two stimulus features (orientation and spatial frequency) within the same neurons, specifically in layer 2/3 excitatory neurons. The majority of neurons that were tracked and tuned on consecutive imaging sessions maintained stable orientation and spatial frequency preferences (83% and 76% of the population, respectively) over a 2-week period. Selectivity, measured as orientation and spatial frequency bandwidth, was also stable. Taking into account all 4 parameters, we found that the proportion of stable neurons was less than two thirds (57%). Thus, a substantial fraction of neurons (43%) were unstable in at least one parameter. Furthermore, we found that instability of orientation preference was not predictive of instability of spatial frequency preference within the same neurons. Population analysis revealed that noise correlation values were stable well beyond the estimated decline in monosynaptic connectivity (~250–300 microns). Our results demonstrate that orientation preference is stable across a range of spatial frequencies and that the tuning of distinct stimulus features can be independently maintained within a single neuron.

## Introduction

The extent to which stimulus tuning features are reliability represented at the level of single neurons long-term, across days remains an open question. On one hand, a number of studies report that stimulus representation within single neurons is generally stable in baseline conditions^1–7^. Consistent with the observation that in primary visual cortex (V1) subthreshold input onto dendritic spines exhibits stable orientation tuning^8^, longitudinal imaging of neuronal soma revealed that in the absence of training, orientation preference is stable, for the low spatial frequency examined^5,6^. On the other hand, studies in both motor and sensory cortex in awake animals report that stimulus-response tuning curves are variable either prior to or during training and become more stable after reinforced learning^9,10^. For example, in baseline conditions orientation selectivity was found to be stable in approximately only 20–50% of neurons tracked in the primary visual cortex (V1) of awake mice tested on two different orientations presented at a single spatial frequency. Orientation selectivity was stabilized after the mice learned to successfully discriminate orientated bars for a reward^11^. Similarly, in motor cortex within-neuron tuning variability was found to decrease after training in primates and rodents^1,12,13^. Training-induced stabilization is also observed in the hippocampus^14,15^. Furthermore, it has been suggested that only a small fraction of the neuronal population needs to maintain feature selectivity to achieve stable perception^2,7^ and to mediate recovery of response selectivity within the local network following perturbation^5,10^. Computational studies have referred to highly stable neurons capable of facilitating network recovery following perturbation as ‘anchor cells’. Taken together, evidence suggests that for low spatial frequency there does exist a stable population of neurons in V1 resistant to perturbation. However, the extent to which these observations apply to higher, behaviorally relevant spatial frequencies is unknown. Furthermore, the extent to which multiple stimulus tuning features are reliability represented in a single V1 neuron is unclear.

To better define the fraction of neurons that consistently respond to spatial frequencies known to be well-represented in mouse V1, we systematically characterized the tuning stability of two stimulus features: orientation and spatial frequency in awake mice. In addition we considered population stability by examining signal and noise correlations. We found that in the absence of any experimental training signals or perturbation, orientation preference is highly stable across a range of spatial frequencies in tuned neurons. Consistent with this observation, we found that cross-session pairwise signal correlations were highly stable; cross-session correlation values were >0.75. These data confirm that in awake mice, orientation preference across a range of spatial frequencies is highly stable in tuned neurons. However when considering a larger parameter space of stimulus response features, our data demonstrate that within a single neuron aspects of stimulus representation can drift while orientation preference remains stable. Our results provide insight into the basis by which stable visual perception is maintained during normal experience and daily interactions with the environment.

## Results

**Figure 1:**
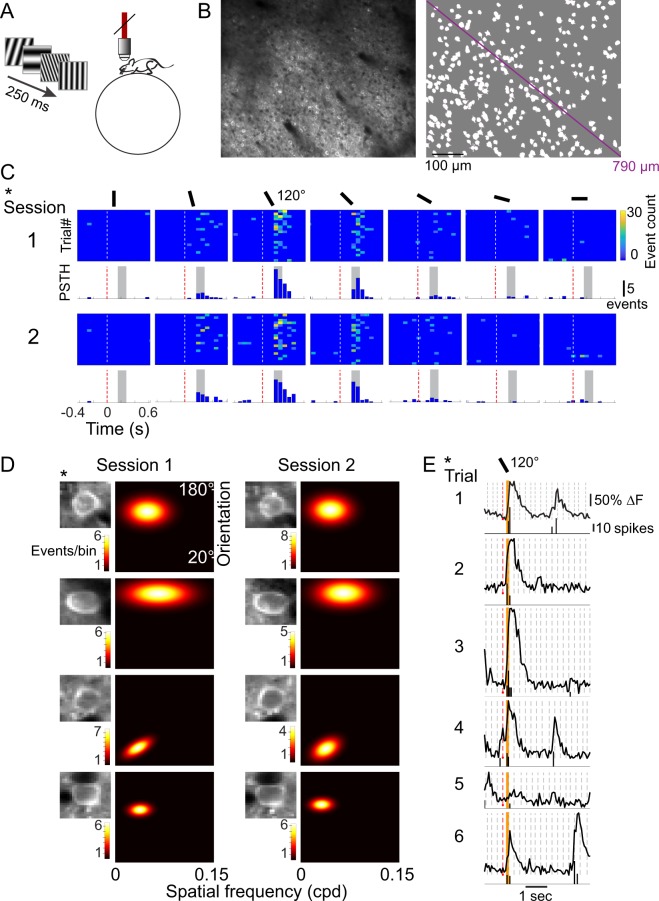
Experimental design and quantification of tuning. (**A**) Experimental Design. Two-photon calcium imaging was performed in awake, head-fixed mice head-fixed atop a floating spherical treadmill. Grating stimuli were shown continuously (4 stimuli/s) on a screen that covered 142 × 96 degrees of the visual angle. (**B**) Mean intensity image (left) and identified neurons (right) from one example session. The imaged field of view was 620 × 504 μm. (**C**) Responses of an example neuron, Session1 (top row) and Session2 (bottom row). Each column shows the responses to one stimulus and the orientation of the stimulus is indicated by the black bar above each column, each row is a single trial. Trials are aligned to the onset of the stimulus (t = 0 seconds, dashed line). Average peri-stimulus time histograms are shown below each panel. The temporal window over which the responses were quantified is indicated in gray. (**D**) Four example tracked neurons and their Gaussian tuning on Session1 (left) and Sesison2 (right). Asterisk indicates the same neuron as in panel C. Event bin duration was 67 ms. Inset shows the mean intensity image of the neuron from the respective session. Dimensions of the insets are 17.8 × 20.6 μm. (**E**) Fluorescence trace and the deconvolved event estimate of the same example neuron as (**C**) for the first 6 trials in Session1. Fluorescence traces are shown on top and the corresponding deconvolved event estimates are shown below the trace. The dashed lines indicate stimuli onsets. The preferred stimulus is indicated by the red dashed line. The orientation of the preferred stimulus is indicated by the black bar above. The window for quantifying the response is shown in orange.

The majority of visually responsive neurons in mouse primary visual cortex (V1) prefer spatial frequencies of 0.15 cycles per degree (cpd) or lower^16^. A previous study examined stability of orientation preference and selectivity specifically at 0.04 cpd in anesthetized mice and found a substantial fraction of neurons were stable in control conditions^5^, and second study in awake mice reported considerable stability of orientation tuning specifically at 0.05 cpd^6^. Our goal was to more precisely define the fraction of persistently tuned neurons in V1 as well as to examine a range of spatial frequencies. Using 2-photon calcium imaging in awake head-fixed transgenic mice expressing GCaMP6f exclusively in excitatory neurons^17^ raised in standard, non-enriched housing conditions, we compared the tuning stability of orientation and spatial frequency within individual neurons and at the population level. Static gratings were presented at varying orientations and spatial frequencies (range, 0.0125–0.15 cpd). Mice were positioned to passively view the stimuli, without reward. To control for behavioral state, we restricted our analysis to trials in which mice were not running. Two-dimensional (2-D) tuning curves were generated to quantify individual neuron responses (Figs 1, S1). A total of 312 stimuli were presented, each for 250 ms, in random order without an intervening gray screen^18,19^. Given that we used a rapid presentation of visual stimuli and the decay kinetics of GCaMP6f are prolonged relative to actual spike events^8^, neuronal signals were deconvolved to estimate spike event times in order to more accurately assign responses to each stimuli (Figs 1E, S2). Low spiking activity likely goes undetected when using calcium imaging in conjunction with deconvolution, so it possible that weakly responsive neurons are scored as non-responsive in our data set. Neurons were segmented and registered across sessions (Fig. S3). Neurons successfully registered across sessions are referred to as ‘tracked’.

**Figure 2:**
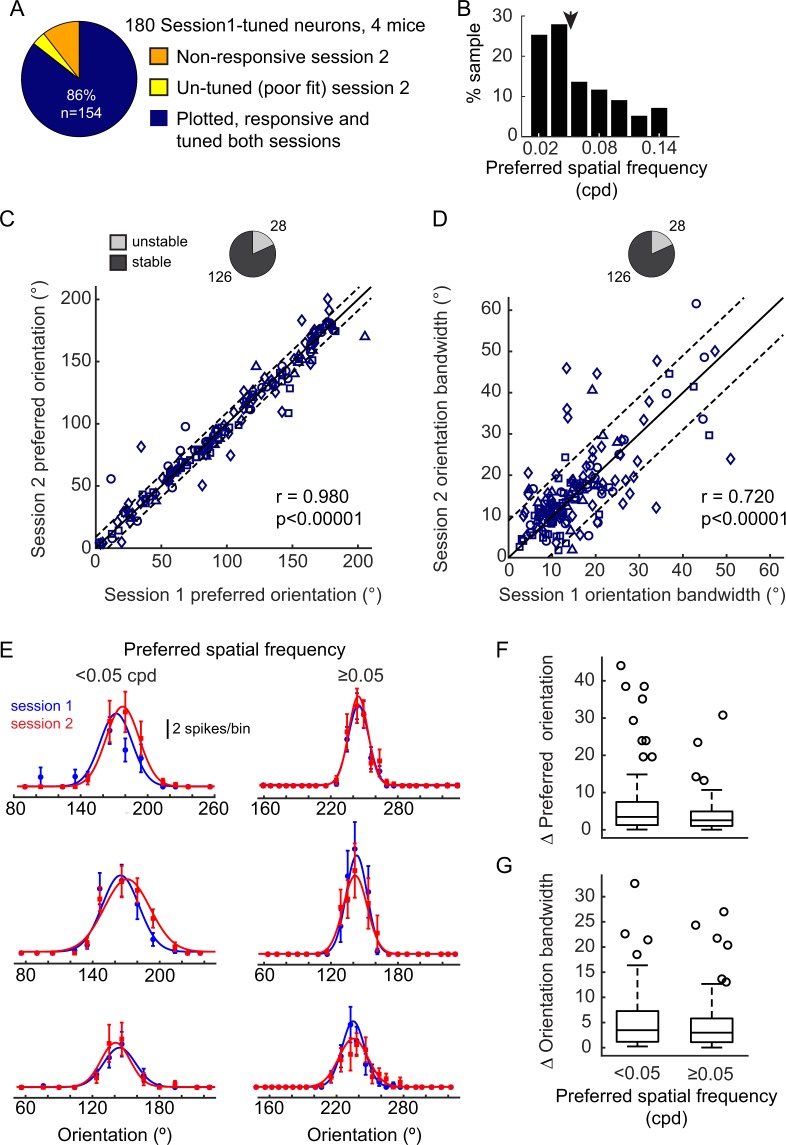
Orientation tuning is stable across a range of spatial frequencies. (**A**) Distribution of non-responsive and reliably responsive neurons tracked for two imaging sessions (6 ± 2 days apart). A total of 180 neurons were responsive and tuned in Session1. To be considered reliably responsive (blue), neurons needed to be well fit by the 2-dimensional Gaussian function and responsive on both sessions. In Session2, 11% of the neurons were non-responsive (orange), and 4% were not well fit by the 2-dimensional Gaussian (yellow). (**B**) Distribution of preferred spatial frequencies of the 180 tracked neurons that were responsive and tuned on Sesison1. The median preferred spatial frequency was 0.05 cycles per degree (arrowhead). (**C**) Comparison of preferred orientation between Session1 and Session2. Solid black line indicates the unity line, dashed lines indicate the median increment of the stimulus space tested (9°). Neurons from different mice are indicated by different symbols. Pearson’s correlation r = 0.980 (p = 1.03e-107). 28/154 neurons (light gray, pie plot) had a shift in the preferred orientation that was larger than 9°. (**D**) Comparison of orientation tuning bandwidths between Session1 and Session2. Lines are drawn with the same criteria as in (**C**). Pearson’s correlation r = 0.720 (p = 6.23e-26). 28/154 neurons (light gray, pie plot) had a shift in bandwidth that was larger than 9 degrees. We noted that 44/154 neurons were unstable in at least one of the two orientation parameters (not shown). (**E**) Three examples of orientation tuning for neurons preferring spatial frequencies < 0.05 cpd (left) and three examples of neurons preferring higher spatial frequencies (right). Mean ± sem, as well as the fitted curves are shown for Session1 (blue) and Sesison2 (red). (**F**) Box and whisker plots show that the difference in preferred orientation between Session1 and Session2 for neurons preferring high spatial frequencies (right) fell well within the range of differences exhibited by neurons preferring spatial frequencies less than 0.05 cpd (left).

**Figure 3:**
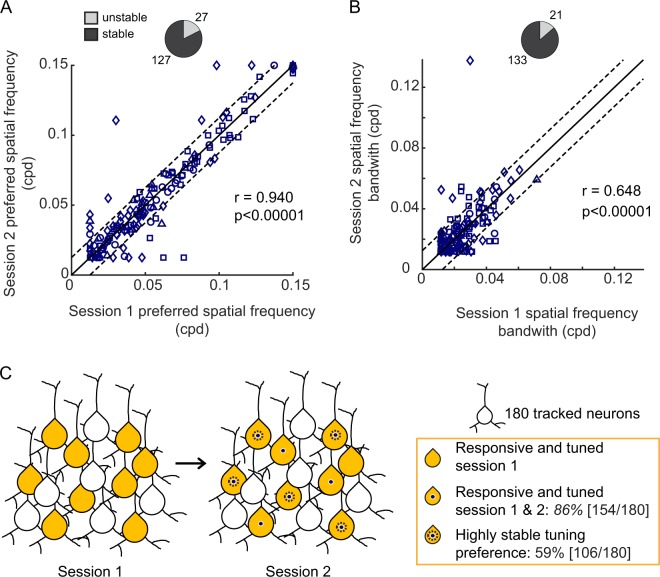
Spatial Frequency tuning is stable. (**A**) Comparison of preferred spatial frequency between Session1 and Session2. Solid black line indicates the unity line, dashed lines indicate the median increment of the stimulus space tested (0.0125 cpd). Symbols and color schemes as in Fig. 2C,D. Pearson’s correlation r = 0.940 (p = 1.21e-72). 27/154 neurons (light gray, pie plot) had a shift in the preferred spatial frequency that was larger than 0.0125 cpd. (**B**) Comparison of spatial frequency tuning bandwidths between Session1 and Session2. Lines are drawn with the same criteria as in (**A**). Pearson’s correlation r = 0.648 (p = 1.13e-19). 21/154 neurons (light gray, pie plot) had a difference in spatial frequency tuning bandwidth that was larger than 0.0125 cpd. We noted that 36/154 neurons were unstable in at least one of the two spatial frequency tuning parameters (not shown). (**C**) Summary of tuning stability across 6 ± 2 days in mouse V1, schematic representation (left) and actual measured percentages (right). Of the responsive and tuned neurons in Session1 (yellow filled), 86% were responsive and tuned in Session2, 59% were highly stable in both orientation tuning preference and spatial frequency tuning preference (69% [106/154] if considering the starting pool to be those neurons that were responsive and tuned in *both* sessions). Note, if considering all 347 tracked neurons, not just the neurons tuned to the presented grating stimuli, 31% [106/347] of the neurons in the population were detected to be stable. Tuning preference as used here refers specifically to preferred orientation and preferred spatial frequency.

**Figure 4:**
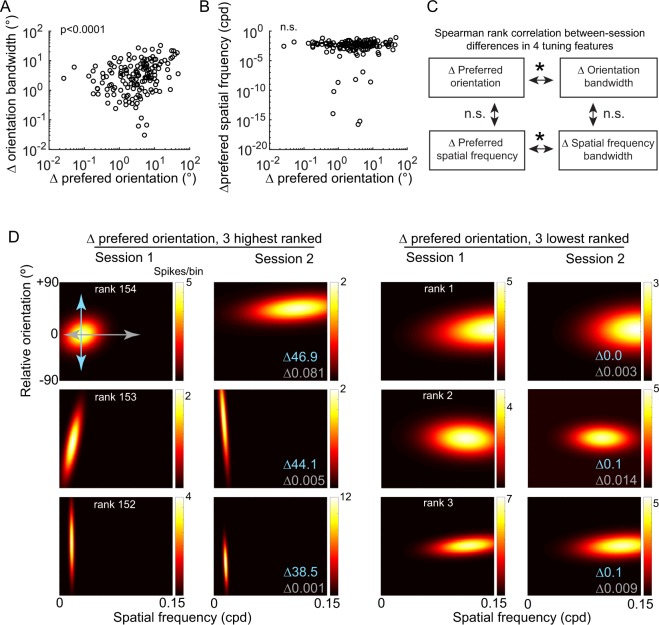
Orientation tuning stability is independent of spatial frequency tuning stability. (**A**) Magnitude of change between Session1 and Session2 in preferred orientation versus orientation bandwidth, Spearman’s Rank correlation r = 0.330, p = 2.85e-5. Data are plotted on a logarithmic scale to more clearly visualize individual data points. (**B**) Magnitude of change between Session1 and Session2 in preferred orientation versus preferred spatial frequency, Spearman’s Rank correlation r = 0.103, p = 0.205. Data are plotted on a logarithmic scale to more clearly visualize individual data points. (**C**) Summary of correlations among differences in the 4 tuning parameters measured. In addition to the relationships described in (**A**,**B**), the difference in preferred spatial frequency was positively correlated to the difference in spatial frequency bandwidth (Spearman’s Rank Correlation r = 0.397, p = 3.43e-7), but the difference in orientation bandwidth was not correlated with the difference in spatial frequency bandwidth (Spearman’s Rank Correlation r = −0.125, p = 0.122). (**D**) Gaussian tuning of 6 example neurons, ranked by magnitude of change in orientation preference. Blue numbers indicate the change in preferred orientation, gray numbers indicate the change in preferred spatial frequency. For the three neurons with the largest shift in preferred orientation (left, rank 154–152), the neuron with the largest shift in preferred orientation also showed a large shift in the preferred spatial frequency, whereas the two neurons with the next largest shifts in preferred orientation did not show a large change in preferred spatial frequency. For neurons with the smallest shift in orientation (right, rank 1–3), one neuron showed a considerable shift in the preferred spatial frequency (second row).

**Figure 5:**
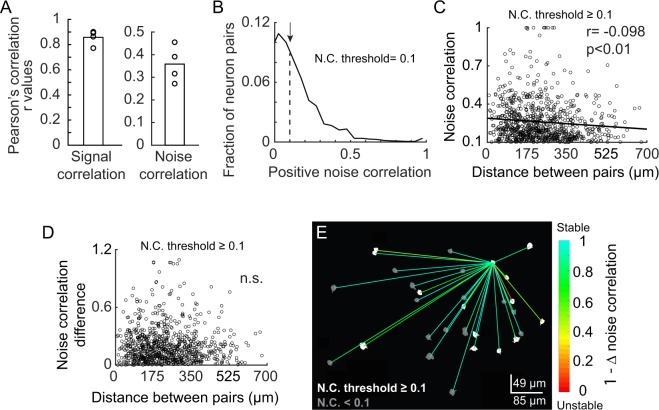
Noise correlation is stable at long distances. (**A**) Similarity of pairwise signal and noise correlation between sessions. Each circle indicates an animal and the bar indicates the mean similarity across animals. (**B**) Distribution of pairwise noise correlation in Session 1. Negative noise correlations are not shown. 25% of all neuronal pairs had noise correlations larger than 0.1. Only these pairs were further analyzed to examine the relationship with distance to remove the effects of neurons with small magnitude noise correlations. (**C**) Relationship between noise correlation and distance between neurons. Pairwise noise correlation slightly decreased as a function of distance (Pearson’s correlation r = −0.098, p = 4.20e-3, n = 852 pairs). (**D**) Relationship between the difference in noise correlation (difference between sessions) and distance between neurons. The difference in noise correlation was not correlated with distance (Pearson’s correlation r = −0.027, p = 0.436). (**E**) Noise correlation difference for an example reference neuron. Neurons with noise correlations larger than or equal to 0.1 in Session1 are shown in white. All other neurons are shown in gray. The colors of the lines indicate the stability of noise correlation between Session1 and 2 measured as 1 − ∆ noise correlation, where ∆ noise correlation is the absolute magnitude of the change in noise correlation. Note, the pair of neurons that are most unstable (red line) are relatively close to one another. Four neurons did not have a measurable noise correlation with the reference neuron and are not shown.

**Figure 6:**
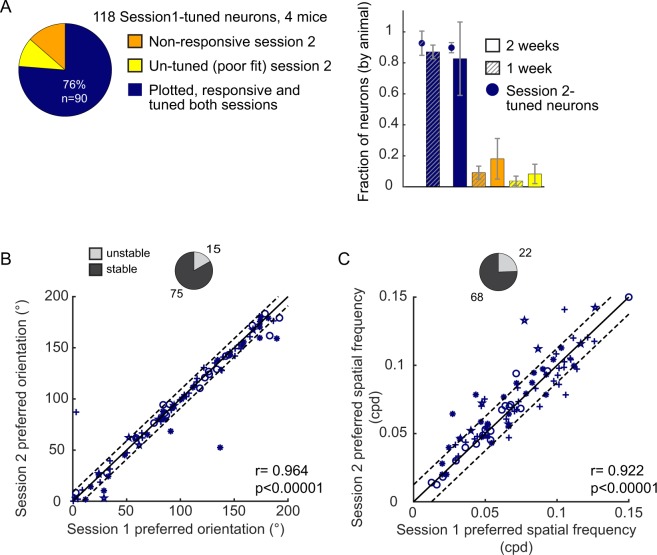
Stability of tuning parameters across 2 weeks. (**A**) Left, distribution of tracked neurons pooled from all 4 animals between two sessions spanning 14 ± 2 days. 118 neurons that were responsive and well-tuned in Session1 were tracked in Session2. 16 (14%) of the tracked neurons were not responsive in Session2 (orange) and 12 (10%) were not well-fit by a Gaussian (yellow). 90 (76%) were responsive and well-fit by a Gaussian in both sessions (blue). Right, means across animals (bars) for the 1 and 2-week data sets. Offset circles, in the 1-week data set (hashed markings), 170 neurons were tuned in Session2, and of those neurons 92.7 ± 0.08% were tuned in Session1. In the 2-week data set 102 neurons were tuned in Session2, and of those neurons 89.8 ± 0.03% were tuned in Session1. Error bars are ± std. (**B**) Comparison of preferred orientation of neurons between sessions spanning 14 ± 2 days. The solid and dashed black lines are identical to Fig. 2C. Pearson’s correlation r = 0.964 (p = 1.88e-52). 15/90 neurons (light gray, pie plot) had a shift in the preferred orientation that was larger than 9 degrees. (**C**) Comparison of preferred spatial frequencies of neurons between sessions spanning 14 ± 2 days. The solid and dashed black lines are identical to Fig. 3A. 22/90 neurons (light gray, pie plot) had a shift in the preferred spatial frequency larger than 0.0125 cpd. Pearson’s correlation r = 0.922 (p = 5.64e-38).

Two data sets were acquired and analyzed. Neurons were imaged across two sessions, spanning the course of one week (Data Set 1: 6 ± 2 days apart, 4 mice, Figs 1–5) and two weeks (Data Set 2: 14 ± 2 days apart, 4 mice, Fig. 6).

### Orientation preference is stable across a range of spatial frequencies

In the first data set, a total of 347 neurons were tracked from 4 mice. 56% (196/347) of the tracked neurons exhibited a response to at least one of the stimuli in our stimulus set on session 1 and/or session 2. We cannot make conclusions regarding the stability of neurons that did not respond in either session, because it is possible that the optimal stimulus was not presented to these neurons. Of the tracked neurons, 180 were responsive and well-fit by a 2-dimensional Gaussian function (tuned) on the first session. Pooled from 4 animals, 86% (154) of the tracked neurons were responsive and tuned on both sessions (Fig. 2), this represents a 14% drop in number of tuned and responsive neurons on session 1 compared to session 2. We examined stability of orientation preference as well as orientation bandwidth across a range of spatial frequencies. For neurons identified as being reliably responsive in both imaging sessions, the preferred orientation of individual neurons on the first imaging session was highly correlated with their preferred orientation on the second imaging session (Pearson’s r = 0.980, p < 0.00001; Fig. 2C). Less than twenty percent of the neurons had a shift in preferred orientation that was larger than 9°, the median increment of the stimulus space tested. Orientation tuning bandwidth was also stable, although there was greater heterogeneity in this feature among neurons in terms of magnitude of shift, compared to orientation preference as indicated by a lower r value (Pearson’s r = 0.720, p < 0.00001; Fig. 2D). In terms of number of unstable neurons, less than 20% had a shift greater than 9°. Twenty-nine percent (44/154) of the neurons exhibited a cross-session change >9° in at least one of the two orientation parameters. To provide a simple summary of the results, we refer to cross-session changes in orientation preference or bandwidth greater than 9° as unstable. Similarly, cross-session changes in spatial frequency preference or bandwidth greater than 0.0125 cpd (the spacing of vertical and horizontal stimulus gratings), are referred to as unstable. To confirm that orientation preference was stable in neurons preferring higher spatial frequencies, we divided the population into two groups (high and low spatial frequency) at the median of the distribution, 0.05 cpd (Fig. 2B). Stability of orientation preference for high spatial frequency-preferring neurons was within the stability range of neurons preferring lower spatial frequencies (Fig. 2E,F). Stability of bandwidth was also overlapping between the two groups (Fig. 2G).

Given our experimental design, we were in a position to examine the stability of spatial frequency preference. Similar to orientation preference, we found that preferred spatial frequency was highly stable (Pearson’s r = 0.940, p < 0.00001; Fig. 3A). Also similar to orientation bandwidth, we found that the bandwidth of spatial frequency tuning was stable. As with orientation tuning, there was greater heterogeneity in the magnitude of shift in bandwidth compared to preference as indicated by a lower r value (Pearson’s r = 0.648, p < 0.00001; Fig. 3B). Taken together, these data confirm that in tuned neurons, individual tuning features are stable in awake mice across a range of spatial frequencies and establish that spatial frequency tuning is stable as well as orientation tuning (Fig. 3C). The proportion of stable neurons is summarized in Table 1.

**Table 1:** Percentages of stable neurons, across 1 week.

1 Week Data	Number of neurons	% of Tracked Neurons	% of Responsive and Tuned in Session 1	% of Responsive and Tuned in Sessions 1&2
All Tracked Neurons	347	—	—	—
Responsive and Tuned in Session1	180	52%	—	—
Responsive and Tuned in Sessions 1&2	154	44%	86%	—
Stable Orientation Pref (∆ ≤ 9°)	126	36%	70%	82%
Stable Orientation BW (∆ ≤ 9°)	126	36%	70%	82%
Stable Orientation Pref and BW	110	32%	61%	71%
Stable SF Pref (∆ ≤ 0.0125 cpd)	127	37%	71%	82%
Stable SF BW (∆ ≤ 0.0125 cpd)	133	38%	74%	86%
Stable SF Pref & BW	118	34%	66%	77%
Stable in SF Pref & Orientation Pref	106	31%	59%	69%
Stable in all 4 parameters	87	25%	48%	56%

We next sought to test whether instability of one feature co-varied with another across the population of tuned neurons (Fig. 4). We found that the magnitude of change in preferred orientation across sessions was significantly correlated with the magnitude of change in orientation bandwidth (Rank Sum r = 0.330, p < 0.0001; Fig. 4A). These data indicate that if unstable in orientation preference, the neuron is more likely to be unstable in orientation bandwidth. We confirmed that this trend holds for neurons with changes in orientation greater than 1 degree (Fig. S4). Note, not all individual neurons exhibited a correlation between these two parameters, as evident in Fig. 2. Some neurons exhibited a shift in orientation preference greater than 9 degrees and did not exhibit a shift greater than 9 degrees in orientation bandwidth. In contrast to orientation preference and orientation bandwidth, the magnitude of change in orientation preference across the population of tuned neurons was not correlated with the magnitude of change in spatial frequency preference (Rank Sum r = 0.103, p = 0.205; Fig. 4B). The relationship of stability across features is summarized in Fig. 4C. These results indicate that the mechanisms regulating stability of preferred orientation are dissociable from those that regulate stability of preferred spatial frequency, within a single neuron.

### Stability of noise correlation does not decrease with distance, measured out to 700 microns

Next we examined neural responses at the population level. As expected from the results shown in Figs 2C,D and 3A,B, pairwise correlation in mean stimulus response (i.e., signal correlation) revealed that signal correlation matrices were highly similar across imaging sessions (average Pearson’s correlation across 4 mice r = 0.867 ± 0.060; Figs 5A, S4). The cross-session similarity in signal correlation found here is consistent with previous observations in anesthetized^5^ and awake^6^ animals, although we find a higher degree of similarity than previously reported.

Correlation in trial-to-trial fluctuations of response magnitude across neuron pairs (i.e. noise correlation) is often used to infer functional connectivity^20–22^. It is generally found that both monosynaptic connection probability and noise correlation decrease as a function of spatial distance^23^. It was previously found that in addition to stability of orientation preference and selectivity, noise correlation among pairs of neurons within a within a 185 × 185 µm field of view is stable in anesthetized mice^5^. Our imaging field of view allowed us to address the extent to which stability of noise correlation dropped off with spatial distance, for distances up to 700 microns (Fig. 5). First, we found that noise correlation was stable in all mice (p < 0.0001). Pearson’s correlation r values ranged between 0.272 to 0.455 (Fig. 5A). The distribution of positive noise correlation values pooled across animals is shown in Fig. 5B. Notably, we found that the stability of noise correlation across the two imaging sessions did not change with distance. The absolute difference in noise correlations values for those pairs of neurons that exhibited positive noise correlation (Δ noise correlation) on at least one imaging session was compared to spatial distance between the neuron pairs (Fig. 5D, Pearson’s correlation r = −0.027, p = 0.436). To visualize the distance versus Δ noise correlation relationship, the spatial location of one reference neuron in relation to all of its pairs is shown in Fig. 5E. These results raise the possibility that common input contributes to the maintenance of population stability beyond what is expected for monosynaptic connectivity, which falls off at distances of 250–300 microns^23^.

### Stability over the course of two weeks

In a second data set, neurons were tracked over the course of two weeks (14 ± 2 days). A total of 330 neurons were tracked in 4 mice. One of the mice from the first data set was included in the second data set. Of the tracked neurons, 118 were responsive and well-fit by a 2-dimensional Gaussian function (tuned) on the first session. Pooled across animals, 76% (90) of the tracked neurons were responsive and tuned on both sessions (Fig. 6A). Overall, the results for the 2-week data set were similar to the 1-week data set. The preferred orientation of individual neurons on the first imaging session was highly correlated with their preferred orientation on the second imaging session (Pearson’s r = 0.964, p < 0.00001; Fig. 6B). Less than twenty percent of the neurons had a shift in preferred orientation that was larger than 9 degrees. Orientation tuning bandwidth was also stable, although there was greater heterogeneity in this feature among neurons in terms of magnitude of shift, compared to orientation preference as indicated by a lower r value (Pearson’s r = 0.777, p < 0.00001). In terms of number of unstable neurons, 8/90 had a shift greater than 9 degrees. Twenty-two percent (20/90) of the neurons were unstable in at least one of the two orientation parameters. Margolis *et al*. (2013)^3^ raise the possibility that weakly selective neurons may be less stable than sharply tuned neurons. We examined our data set, and found that stability of orientation preference did not correlate with orientation tuning bandwidth (Pearson’s r = 0.0489, p = 0.647). Similar to the 1-week data set, preferred spatial frequency on the first imaging session was highly correlated with the preferred orientation on the second imaging session (Pearson’s r = 0.922, p < 0.00001; Fig. 6C), and the bandwidth of spatial frequency was also stable (Pearson’s r = 0.701, p < 0.00001). The proportion of stable neurons is summarized in Table 2. We noted that in the 1-week data set 14% of the neurons responsive on session 1 were not responsive on session 2 (pooled from all 4 animals). The mean across animals was 12.9 ± 2% (±sem). Similarly, the mean across animals in the 2-week data set was 16.1 ± 12% (±sem). There was a similar fraction of neurons that were responsive and tuned in session 2 only (in other words, not tuned and/or responsive in session 1), in both the 1 and 2-week data sets (Fig. 6A). Thus, there was not a systematic loss of responsiveness at the population level over time.

**Table 2:** Percentages of stable neurons, across 2 weeks.

2 Week Data	Number of neurons	% of Tracked Neurons	% of Responsive and Tuned in Session 1	% of Responsive and Tuned in Sessions 1&2
All Tracked Neurons	330	—	—	—
Responsive and Tuned in Session1	118	36%	—	—
Responsive and Tuned in Sessions 1&2	90	27%	76%	—
Stable Orientation Pref (∆ ≤ 9°)	75	23%	64%	83%
Stable Orientation BW (∆ ≤ 9°)	82	25%	69%	91%
Stable Orientation Pref and BW	70	21%	59%	78%
Stable SF Pref (∆ ≤ 0.0125 cpd)	68	21%	58%	76%
Stable SF BW (∆ ≤ 0.0125 cpd)	80	24%	68%	89%
Stable SF Pref & BW	64	19%	54%	71%
Stable in SF Pref & Orientation Pref	68	21%	58%	76%
Stable in all 4 parameters	51	15%	43%	57%

In terms of estimating the fraction of neurons that could serve as potential anchor cells, if considering only the most stable neurons, our results indicate that approximately one quarter (23%) of the neurons that were tracked are potential candidates for the single feature of orientation preference. On the other hand, the proportion of neurons available to serve as anchor cells simultaneously for all 4 measured features across a 2 week span is estimated to be 15% (51/330).

## Discussion

The response properties of a neuron are largely determined by its synaptic input. Synapses themselves are not static structures. Even in the adult, synapses continuously change their size and strength, and are composed of liable proteins, which turnover on time scales of hours to days^24^. In addition, postsynaptic dendritic spines display structural turnover, in the absence of training signals^25^. In the visual cortex approximately 1% of dendritic spines turn over on a daily basis^26^. However, neural networks comprised of these unstable components are capable of producing reliable stimulus-response output. Reliable output can emerge at the neuronal population level^27–29^, or alternatively exist at the level of single neurons^10^. To clarify the extent to which stability of single neuron response properties may contribute to reliable visual processing, we systematically examined stability of response tuning to both spatial frequency and orientation stimulus features in primary visual cortex of awake mice.

We confirmed that orientation preference and selectivity are largely stable and demonstrated that this stability is true for higher spatial frequencies, as well as low spatial frequencies which was previous observed^5,6^. Taking into account all four tuning parameters (preferred orientation, orientation bandwidth, preferred spatial frequency, and spatial frequency bandwidth) simultaneously in individual neurons, we found that less than half of the tuned neurons were identified as exhibiting high stability in all four parameters over the time span of two weeks. Furthermore, the magnitude of change in orientation preference was not predictive of change in spatial frequency preference. This independence suggests that the mechanisms determining stability of preferred orientation are dissociable from those that determine stability of preferred spatial frequency, within a single neuron. It will be of interest to take this into account in perturbation-recovery models such as those described by Clopath *et al*.^10^.

Given that inhibitory neurons are reported to be more stable than excitatory neurons^1,7,28^, we avoided confounds introduced by cell-type differences as well as depth by targeting our imaging specifically to excitatory neurons within a restricted depth of layer 2/3 (220–260 microns from the pia surface). This specificity may have contributed our finding of higher stability in signal correlation compared to previous reports. In addition, we used static gratings, devoid of directional information. This later difference across the studies raises the possibly that the representation of direction is more flexible. Indeed, studies have demonstrated that just a few hours of passive exposure are sufficient to alter direction selectivity, even in anesthetized conditions^30^.

To examine the relationship between response properties and local network connectivity, a seminal study combined *in-vivo* calcium imaging in anesthetized animals with *in-vitro* electrophysiological slice recordings and was able to characterize the synaptic connectivity profiles of neurons for which response tuning to natural scenes was determined *in-vivo*^31^. This work uncovered a fundamental wiring rule: V1 neurons receive weak synaptic input from many presynaptic partners with diverse response properties and strong input from a few presynaptic partners that share similar response properties. The strong recurrent excitation among similarly tuned ensembles contributes to a neuron’s selectivity by specifically amplifying a particular visual feature^31^. Given this basic architecture, together with our results as well other observations that there is measureable drift in tuning^5,6^, it is interesting to speculate that the relative contribution of strong, specifically tuned input versus weak, diffusely tuned input driving a neuron to spike threshold is subject to drift in baseline conditions.

The ability to longitudinally track the activity of individual neurons using GCamp6 as well as other genetically encoded indicators, has enabled stability to be examined using optical methods, in primary sensory cortex^5,6,32^, association cortex^29^, motor cortex^1,13^, and hippocampus^27,33^. Prior to the availability of optical approaches, there are a few cases in which stability was examined using electrophysiological methods, and these studies are largely in agreement. For example, a study using electrophysiological recording in primates found that orientation preference is stable across days^34^. Some discrepancies do remain between the two techniques, which could in part be related to sampling bias differences^9^. Taken together, an emerging principle from these longitudinal studies is that association areas such as posterior parietal cortex^29^ as well as the hippocampus^15,27,33,35^ exhibit a large degree of instability at the individual neuron level, yet maintain reliable stimulus-response output at the population level. On the other hand, response preference of individual neurons, such as whisker^3^, eye^5^, or orientation preference^5,6^ in primary sensory cortices is stable. Our finding that tuned neurons are highly stable in their orientation preference, across a range of spatial frequencies is consistent with this emerging principle. Furthermore, our data demonstrate that although stimulus representation of a single feature in primary cortex is stable at the level of individual neurons, when considering the representation of multiple stimulus features, the majority of neurons display drift under baseline conditions.

## Methods

### Animals and surgery

All experimental procedures were compliant with the guidelines established by the Institutional Animal Care and Use Committee of Carnegie Mellon University and the National Institutes of Health, and all experimental protocols were approved by the Institutional Animal Care and Use Committee of Carnegie Mellon University. To express the calcium indicator GCaMP6f ^8^ selectively in excitatory neurons^36^, homozygous Emx1cre mice (Jackson Laboratories, stock number 005628) were crossed with homozygous Ai93/heterozygous Camk2a-tTA mice (Jackson Laboratories, stock number 024108). Experimental mice were heterozygous for all three alleles. Mice were housed in a 12 hour/12 hour light cycle and all imaging was performed at Zeitgeber time (ZT) 14.5 ± 1, where ZT0 is lights on, and ZT 12 is lights off. Mice had ad libitum access to food and water. Each experimental mouse was housed with at least one other companion mouse. The same enrichment materials were provided in all cages, and included a Plexiglas hut and nesting material. The first data set consisted of 4 mice (3 females, 1 male) that were each imaged in two sessions spanning 6 ± 2 days. The second cohort of mice consisted of 3 different mice (3 males) that were imaged in two sessions spanning 14 ± 2 days, and one of the mice from the first cohort (1female). This mouse was imaged for a third imaging session 7 days after the second imaging session, thus the first and third image were 14 ± 2 days apart and used in the 2 week data set. A total of 7 mice (3 females, 4 males) were imaged in this study.

Mice (27–45 days old) were anesthetized with isoflurane (3% induction, 1–2% maintenance). A stainless steel bar, used to immobilize the head for recordings, was glued to the right side of the skull and secured with dental cement. A 3-mm diameter craniotomy was made over the primary visual cortex in the left hemisphere, identified by coordinates and landmarks as described^37^. The craniotomy was then covered with a double glass assembly in which the diameter of the inner glass was fitted to the craniotomy and sealed with dental cement. After 2 days of recovery from surgery, the area of the craniotomy and primary visual cortex were confirmed using intrinsic optical imaging as described^38,39^. For all mice, imaging was performed in the binocular zone of the primary visual cortex, confirmed by the location of the imaging field of view with respect to the border between primary visual cortex and higher order visual cortices.

### Data Acquisition

Two-photon calcium imaging was performed in awake head-fixed mice mounted atop a floating spherical treadmill using a resonant scanning microscope (Neurolabware) outfitted with a 16x Nikon objective (0.80 NA). A laser excitation wavelength of 920 nm was used (Coherent, Inc.); green emissions were filtered (Semrock 510/84–50), amplified (Edmund Optics 59–179), and detected with a PMT (Hamamatsu H1 0770B-40). The imaged field of view was 620 × 504 microns, pixel dimensions were 0.85 × 0.98 µm, and the acquisition rate was 15.5 Hz. Treadmill motion was recoded using a camera (Dalsa Genie M640-1/3) synchronized to the resonant scanning mirror; images were sampled at 30 Hz.

### Treadmill Motion Analysis

The behavioral state of animals was classified based on the motion of the treadmill. After applying a threshold on the luminance intensity of the treadmill motion images, phase correlation was computed between consecutive frames to estimate the translation between the frames. The estimated translation was converted to movement speed, taking into account the dimensions of the treadmill motion images (7.8 cm × 7.8 cm)and the acquisition rate of the camera (30 Hz). To align ball motion data with calcium imaging data, the raw movement speed was down-sampled to match the acquisition rate of calcium imaging. To define a motion threshold, the data were smoothed using a 1 s sliding window. Any continuous non-zero movement periods during which the animal’s instantaneous running speed exceeded 10 cm/s threshold for at least one frame were marked as running epochs (Fig. S5). Neural analysis was restricted to non-running trials. The 2-week data set was also analyzed with the motion threshold set to threshold 1.2 cm/s, the stability results were similar (K-S test p values comparing the distribution of cross-session changes in the 4 parameters: 0.80, 0.30, 0.42, and 0.59 Δ preferred orientation, Δ orientation bandwidth, Δ preferred spatial frequency, Δ spatial frequency bandwidth, respectively).

### Data processing: alignment, segmentation, and signal extraction

The acquired image time series were motion-corrected, and individual neurons were segmented in Suite2p toolbox^40^. Motion correction was done by computing the horizontal and vertical translation for each frame using phase correlation. Segmentation was accomplished by first training the classifier included in Suite2P with 30 example imaging sessions which contained approximately 40,000 segments in total. Each session contained approximately 35000 frames acquired at 15.5 Hz, covering a field of view 620 × 504 microns in area. To train the classifier, 4 different users manually labeled segments as either ‘neuron’ or ‘non-neuron’, this was accomplished by examining the mean intensity image and raw fluorescent trace of each of the 40,000 segments. The trained classifier was then used to calculate the probability that a given segment was a neuron. Segments with a calculated probability of >0.6 were labelled as neurons. The user manually inspected segments with a calculated probability in the range of 0.4–0.6 and assigned the label based on shape of segment and mean intensity image of the segment.

Amplitude of calcium transients was expressed in units of inferred spike events using Suite 2p toolbox; in this manuscript we refer to an inferred spike as an ‘event’, e. For each segment n, events e_*n*_ were inferred from fluorescence f_*n*_ using the following model:

$${{\rm{f}}}_{n}={{\rm{e}}}_{n}\ast {\rm{k}}+{\beta }_{n}{{\rm{p}}}_{n}+{{\rm{b}}}_{n}$$

where k is the temporal kernel and b_*n*_ is the baseline fluorescence. A single kernel was derived for all neurons in one imaging session, all other parameters were unique to each individual neuron. Neuropil, which is a contamination of the fluorescence signal f_*n*_ from out of focus cell bodies and nearby axons and dendrites, is modeled by two parameters: p_*n*_, the time course of the neuropil contamination, and *β*_*n*_, the scaling coefficient. * denotes convolution. Using this model, e_*n*_, k, *β*_*n*_, and b_*n*_ were estimated by a matching pursuit algorithm with L0 constraint, in which events were iteratively added and refined until the threshold determined by the variance of the signal was met.

∆F is calculated as,

$${\rm{\Delta }}F=({{\rm{f}}}_{n}-{\beta }_{n}{{\rm{p}}}_{n})-{\rm{median}}({{\rm{f}}}_{n}-{\beta }_{n}{{\rm{p}}}_{n})$$

where f_*n*_ is the raw fluorescence.

A closer inspection of a subset of inferred events revealed that some low amplitude events were likely noise and not true calcium transients, indicating a need to calibrate the Suite 2p toolbox to our data set. To calibrate, we defined a signal threshold. Event-triggered averaging was performed on neuropil subtracted calcium signals, binned by the number of events (0–2, >2–4, >4–6, >6–8, >8–10 events). The averaged calcium trace of low amplitude events had a characteristic negative transient prior to the inferred event time. To systematically remove false positive events, we examined the derivative of the average trace, 0 to 134 ms prior to the event time for each bin. Amplitudes larger than 6 events did not have this negative transient. Therefore, we rejected inferred event amplitudes of less than 6 events as false positives. Output from this calibration is shown in Fig. S2. Segments with at least one event during the course of the imaging session (after applying the 6-event threshold) were considered to be neurons.

### Identification of repeat imaged neurons

To identify neurons that were tracked across imaging sessions, we registered the two imaging sessions for each mouse using the mean intensity image of each session. The mean intensity image for a session was computed by averaging the intensity of each pixel in the aligned calcium image series across time for the entire imaging session (roughly 30000 frames). Then, the mean intensity images of the two sessions were registered, and a rigid transform (X and Y translation and rotation) was computed for the pair of images using one-plus-one evolutionary optimizer. Once the sessions were registered, the percentage of pixel overlap between the segments from two sessions was computed. Segments were accepted to be the same neuron across sessions, if the percentage of overlapping pixels with respect to both the segment from the first session and the segment from the second session was larger than 75%. On average there were 160 pixels in a given segment (Fig. S3).

### Visual Stimulation

Hartley stimuli^18^ were generated in real time using Psychophysics Toolbox (see http://psychtoolbox.org) in Matlab (Mathworks, Boston, MA). The stimulus was presented on a screen positioned 25 cm away from the right eye (contralateral to the imaged hemisphere) angled at 50 degrees with respect to the midline of the animal. The size of the screen was 64 cm by 40 cm, thereby subtending 142 × 96 degrees of visual angle. The maximum spatial frequency was 0.15 cycles/°, which corresponds to 12 cycles along the vertical extent of the display. The Hartley set consisted of the following gratings:

$${H}_{{k}_{x},{k}_{y}}=\pm \,{\rm{cas}}(\frac{2\pi ({k}_{x}x+{k}_{y}y)}{M})$$

where cas(x) ≡ cos(x) + sin(x) and the wavenumbers kx and ky represent the number of cycles along the horizontal and vertical axes, respectively, and these indices ran between −12 and 12, excluding the origin (kx, ky) = (0, 0). Therefore, the total number of different images in the Hartley set was ((2 × 12 + 1)^2^ − 1) × 2 = 1248. The responses to the four spatial phases at each combination of orientation and spatial frequency were averaged, leading to 312 locations in the orientation and spatial frequency domain. Each grating was presented for 250 ms in a consecutively in a random order. Each combination of orientation and spatial frequency was shown at least 20 times in each session. For a subset of mice imaged across the 2-week interval, gratings with even spacing in both spatial frequency and orientation were shown.

### Quantification of visual responses

We used a reverse correlation method^19^ to determine the amplitude of the response. The delay of the analysis window relative to the onset of the stimulus was determined by computing the timing of the peak in the stimulus-averaged events. The peak in the stimulus-averaged events was observed 194 ms to 320 ms after the stimulus was presented on the screen. Therefore, for each stimulus, the response was computed by averaging the number of events between 194 ms and 320 ms window.

For the purpose of this study, we defined a neuron to be responsive to visual stimuli when the number of events following a presentation of a visual stimulus was modulated by the stimuli presented. To test for modulation, we performed a one-way analysis of variance (ANOVA) on the event activity using stimuli as the factor for each neuron, significance was set to α = 0.01.

To estimate the preferred orientation and spatial frequency, and the respective bandwidths of responsive neurons, a two-dimensional Gaussian model was fit using nonlinear least-squared regression such that the number of events R as a function of the orientation θ and the spatial frequency φ of the stimulus is

$$R(\theta ,\phi )=\frac{A}{2\pi {\sigma }_{\theta }{\sigma }_{\phi }\sqrt{1-{\rho }^{2}}}{e}^{(-\frac{1}{2{(1-\rho )}^{2}}[\frac{{(\theta -{\mu }_{\theta })}^{2}}{{\sigma }_{\theta }^{2}}+\frac{{(\phi -{\mu }_{\phi })}^{2}}{{\sigma }_{\phi }^{2}}-\frac{2\rho (\theta -{\mu }_{\theta })(\phi -{\mu }_{\phi })}{{\sigma }_{\theta }{\sigma }_{\phi }}])}+B$$

where μ_θ_ is the preferred orientation and μ_φ_ is the preferred spatial frequency of the stimulus, and the σ_θ_ and σ_φ_ describe the widths of respective tuning. The lower bound for μ_φ_ was set at the lowest spatial frequency of the stimulus, which was 0.0125 cycles per degree. The lower bound for σ_φ_ was set at 0.001 cycles per degree. The covariance of responses for orientation and spatial frequency is captured by the correlation term ρ. A is a parameter accounting for the amplitude of the responses, B is the baseline event activity of the cell. Significance of the fit was assessed by a permutation test, α = 0.05; the 95^th^ confidence interval was calculated by shuffling the stimulus labels of the trial-averaged responses (1000 permutations). In total 312 stimuli were shown. In rare cases, neurons that responded to 3 or fewer stimuli (sharply tuned), despite having high response reliability, did not pass the permutation test. These sharply tuned neurons with high response reliability were included in the analysis.

The bandwidths of the Gaussian tuning were described using half-width at half-maximum (HWHM). The HWHM bandwidths for both orientation and spatial frequency were calculated by

$${\rm{BW}}=\sqrt{2\ast \,\mathrm{ln}(2)}\ast \sigma $$

where *σ* is the width parameter of the Gaussian fit. A small number of neurons were broadly tuned in orientation (BW_orientation_ > 60 degrees). Because orientation parameters of these neurons are not well described by the Gaussian fit, these neurons were removed from analysis.

### Computing Correlations

Signal correlation ρ^sig^ between a pair of neurons is defined as Pearson’s correlation between the average responses to stimuli^41^. Therefore, we computed pairwise signal correlation between neuron i and neuron j as

$${{\rm{\rho }}}_{{\rm{i}},{\rm{j}}}^{{\rm{sig}}}=\mathrm{corr}({\bar{R}}_{i},{\bar{R}}_{j})$$

where $\bar{R}$ is a vector of average response in number of events to each of the 312 sinusoidal gratings for the respective neuron.

Noise correlation *ρ*^noise^ between a pair of neurons is the average Pearson’s correlation between the mean-subtracted single-trial responses. Noise correlation was computed as

$${\rho }_{{\rm{i}},{\rm{j}}}^{{\rm{noise}}}=\frac{{\sum }_{k=1}^{N}{\rm{corr}}({\bar{r}}_{k,i}-{{\rm{R}}}_{k,i},{\bar{r}}_{k,j}-{{\rm{R}}}_{k,j})}{N}$$

where k is the stimulus index, and $\bar{r}$ is a vector of individual responses for the respective neuron and the stimulus. N is the total number of stimuli.

### Statistics

To examine the stability of tuning parameters, Pearson’s correlation was used because it was expected that the comparison of the same tuning parameter between the two sessions would follow a linear relationship. For the same reason, Pearson’s correlation was used to compute the similarity of correlation matrices. However, this assumption may not be true when comparing across different tuning parameters. Therefore, Spearman’s rank correlation was used instead when comparing across different tuning parameters.

### Doxycycline treatment

During the course of this study, the presence of aberrant, large-scale oscillations in the triple-allele transgenic mice that we used was reported^42^. Given that large-scale oscillations could impact our interpretation of stability, we addressed this issue mid-study in the following manner. (1) We noted that no mice used in this study had detectable oscillations in the calcium signal, neither in V1 nor the region anterior to V1 contained within the 3-mm diameter window. (2) We did detect one mouse in our colony, not included in this study, which displayed low frequency oscillations in the recorded calcium signal. Out of approximately 20 mice with windows, just the one had detectable oscillations. Although we do not know for certain why our number of mice with oscillations is lower than reported^42^, a contributing factor could be that our mice are housed in low density conditions and are typically handled by one caretaker. In this rearing regime, mice are less stressed and are less prone to seizures early in life. (3) Upon learning about the oscillation issue, we immediately started treating our female breeders with doxycycline and maintained mice on doxycycline as recommended^42^. One of the mice in the 2-week stability cohort (Fig. 6) was treated. Consistent with previous finding, mice treated with doxycycline until postnatal day (P) 12 did not show GCaMP6f expression at P15 (Fig. S6). Initially introduction of doxycycline to the food supply did reduce the survival rate of the litters, but survival recovered by the third litter after doxycycline introduction: 9/11 (81.2%) dams had their first litter die, while only 1/8 had their second litter die, and 8/8 had viable litters after the third litter. We compared the 2-week stability results between the doxycycline treated mouse and those that did not receive the treatment. The stability of the tuning parameters measured in the treated mouse did not differ from those of untreated mice (two-sample KS test, Δ preferred orientation: p = 0.577, Δ preferred spatial frequency: p = 0.974, Δ orientation tuning bandwidth: p = 0.762, Δ spatial frequency tuning bandwidth, p = 0.458). In addition, signal correlation similarity from the treated mouse was comparable to signal correlation similarity of the untreated population (treated: r = 0.833; untreated: r = 0.786 ± 0.10). Finally, the percentage of neurons that remained responsive and tuned across two weeks was not different between the treated and untreated population (treated: 79.5%; untreated: 74.0 ± 19%).

## Electronic supplementary material

Supplementary Information.
