# Supplementary material for: Feature selectivity is stable in primary visual cortex across a range of spatial frequencies
Source: Sci Rep. 2018 Oct 16;8:15288. doi: 10.1038/s41598-018-33633-2 (PMC6191427; doi:10.1038/s41598-018-33633-2)
Supplement: Supplementary file 1 — Supplementary Information. [file 41598_2018_33633_MOESM1_ESM.pdf]

Feature selectivity is stable in primary visual cortex across a range of spatial frequencies

Brian B. Jeon[1,2], Alex D. Swain[3], Jeffrey T. Good[4], Steven M. Chase[1,2], and Sandra J. Kuhlman[1,2,3,4*]

1. Center for Neural Basis of Cognition, Carnegie Mellon University
2. Department of Biomedical Engineering, Carnegie Mellon University
3. University of Pittsburgh Integrative Systems Biology Program
4. Department of Biological Sciences, Carnegie Mellon University

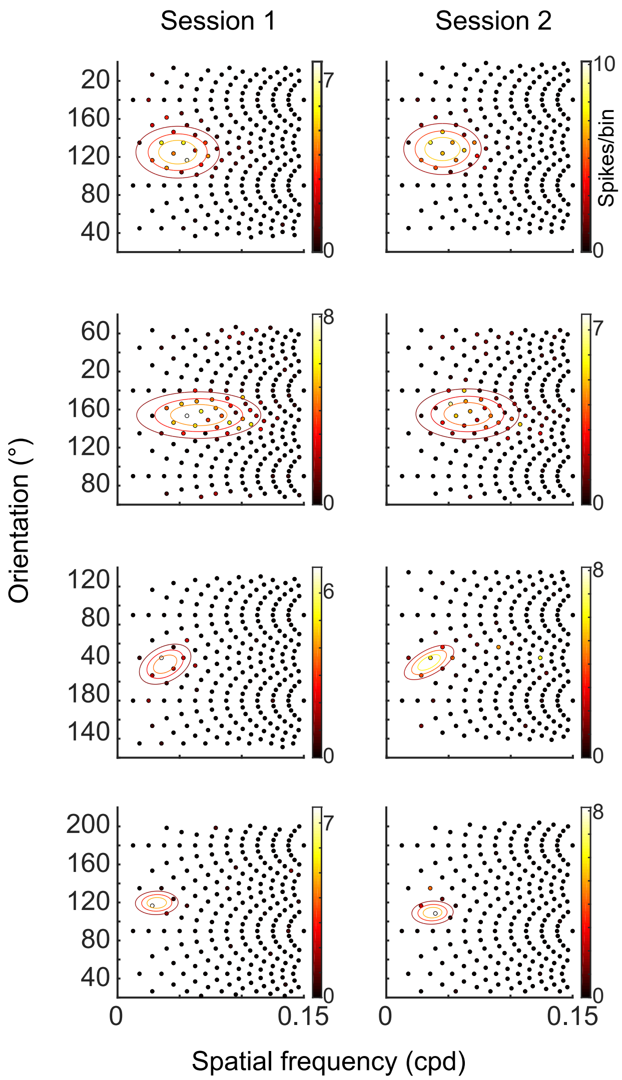

Session 1
Session 2
Orientation (°)
Spatial frequency (cpd)
Spikes/bin
0
0.15
20
160
120
80
40
60
180
140
200
7
10
8
6

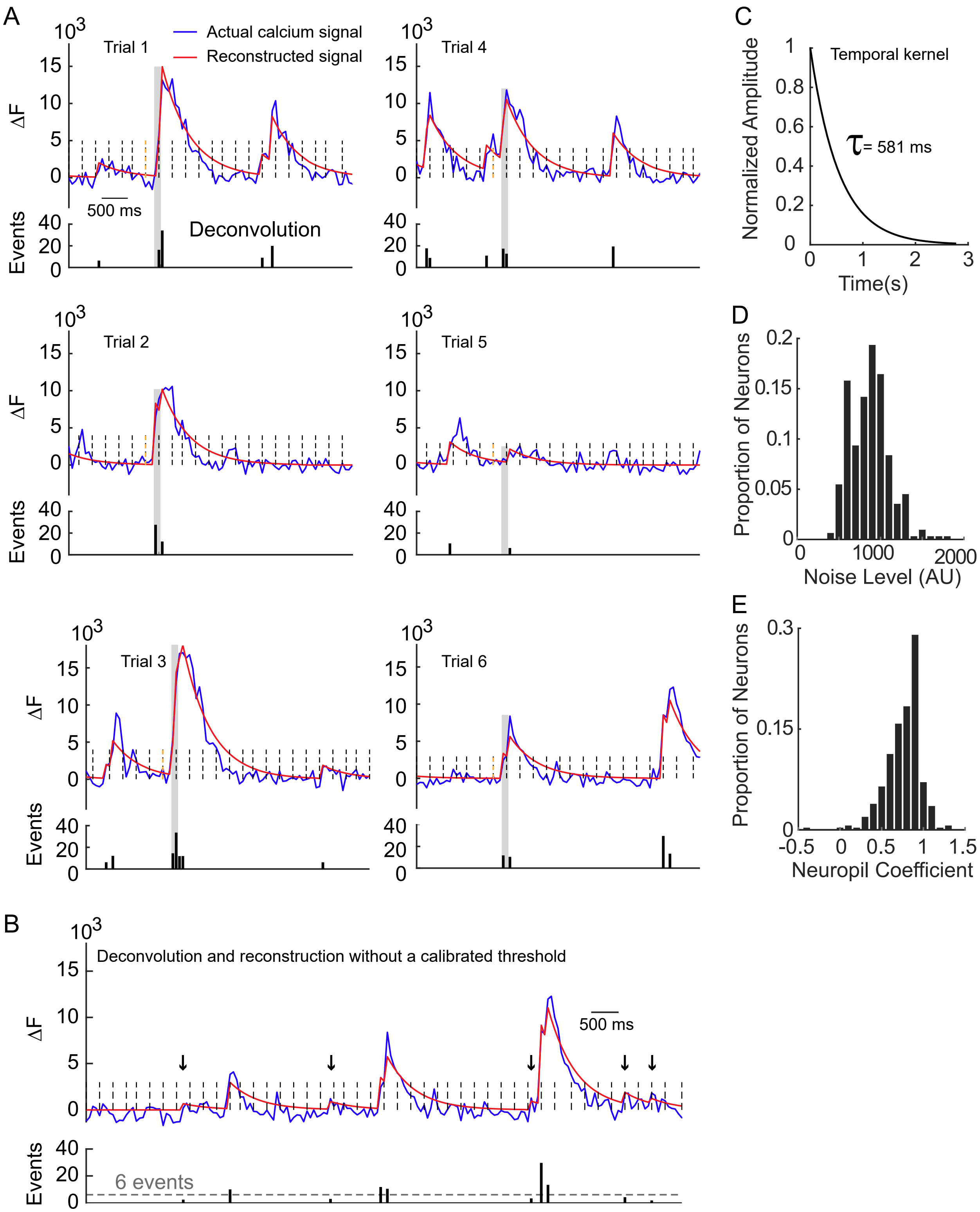
A
Actual calcium signal
Reconstructed signal
Trial 1
Trial 2
Trial 3
Trial 4
Trial 5
Trial 6
ΔF
Events
500 ms
Deconvolution
B
Deconvolution and reconstruction without a calibrated threshold
6 events
C
Temporal kernel
Normalized Amplitude
τ= 581 ms
Time(s)
D
Proportion of Neurons
Noise Level (AU)
E
Neuropil Coefficient

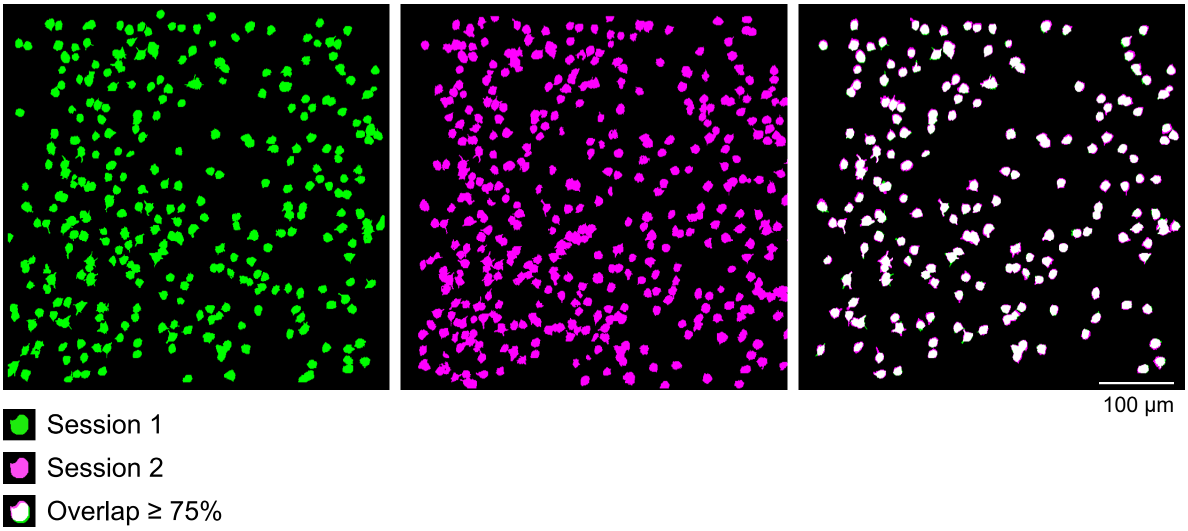
100 μm
Session 1
Session 2
Overlap ≥ 75%

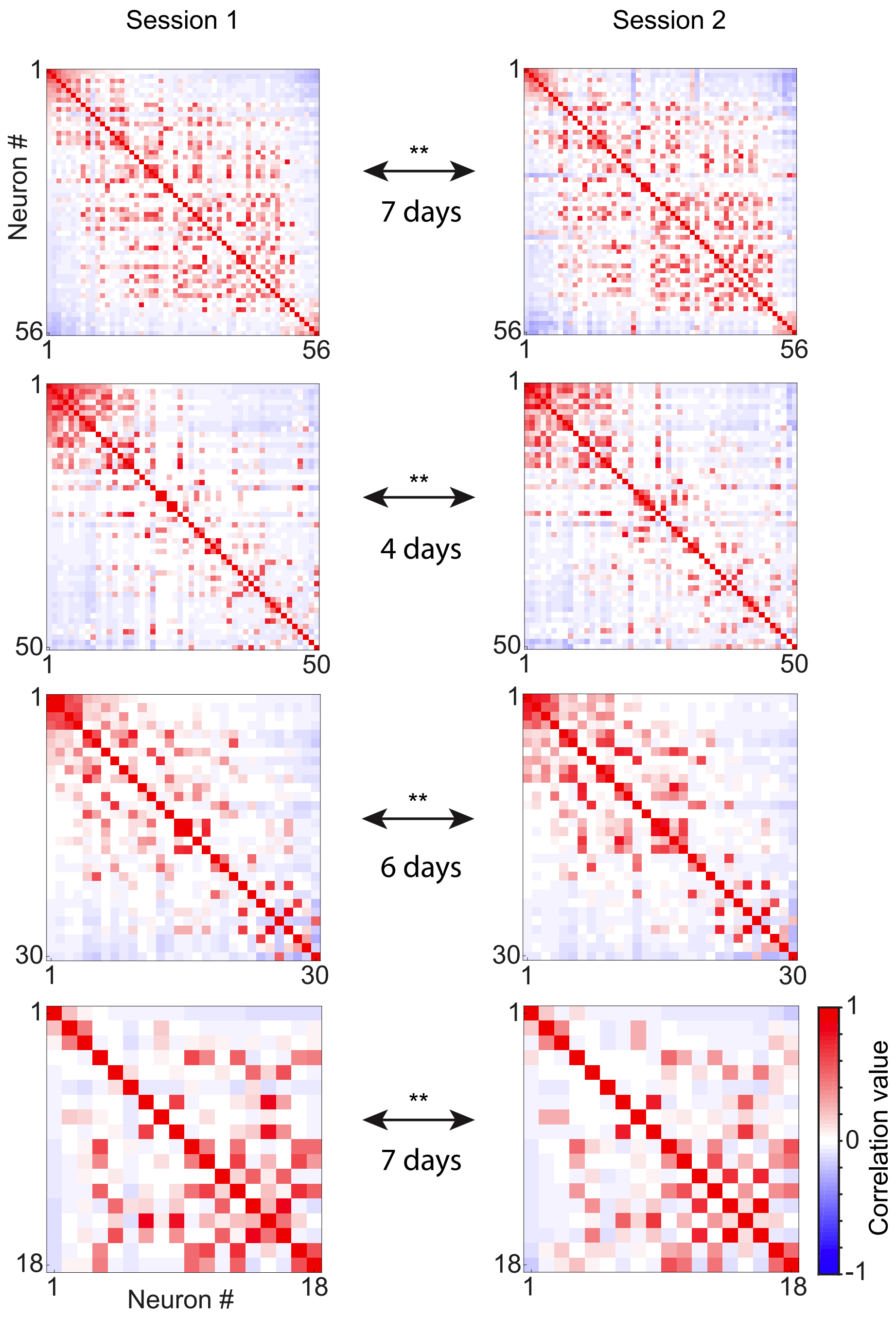
Session 1
Session 2
Neuron #
**
7 days
**
4 days
**
6 days
**
7 days
Neuron #
Correlation value
1
0
-1

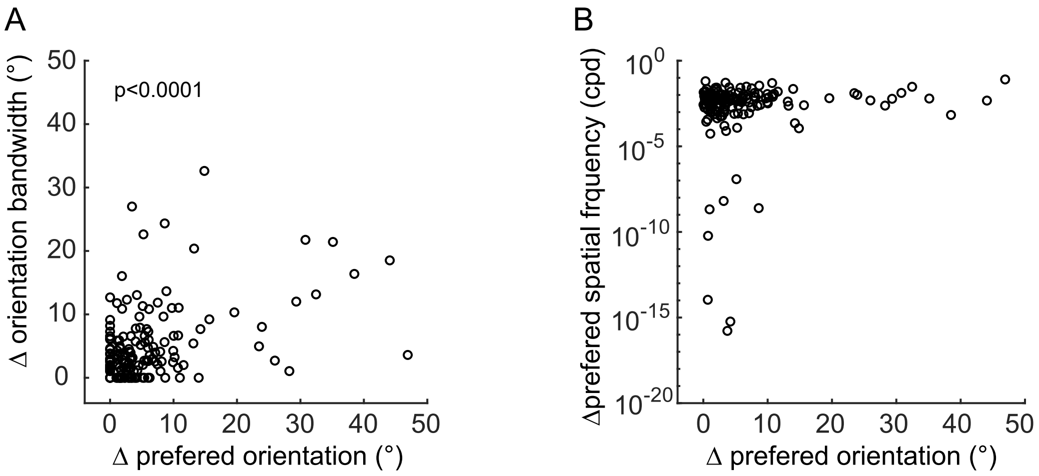
A
p<0.0001
Δ orientation bandwidth (°)
Δ prefered orientation (°)
B
Δprefered spatial frquency (cpd)
Δ prefered orientation (°)

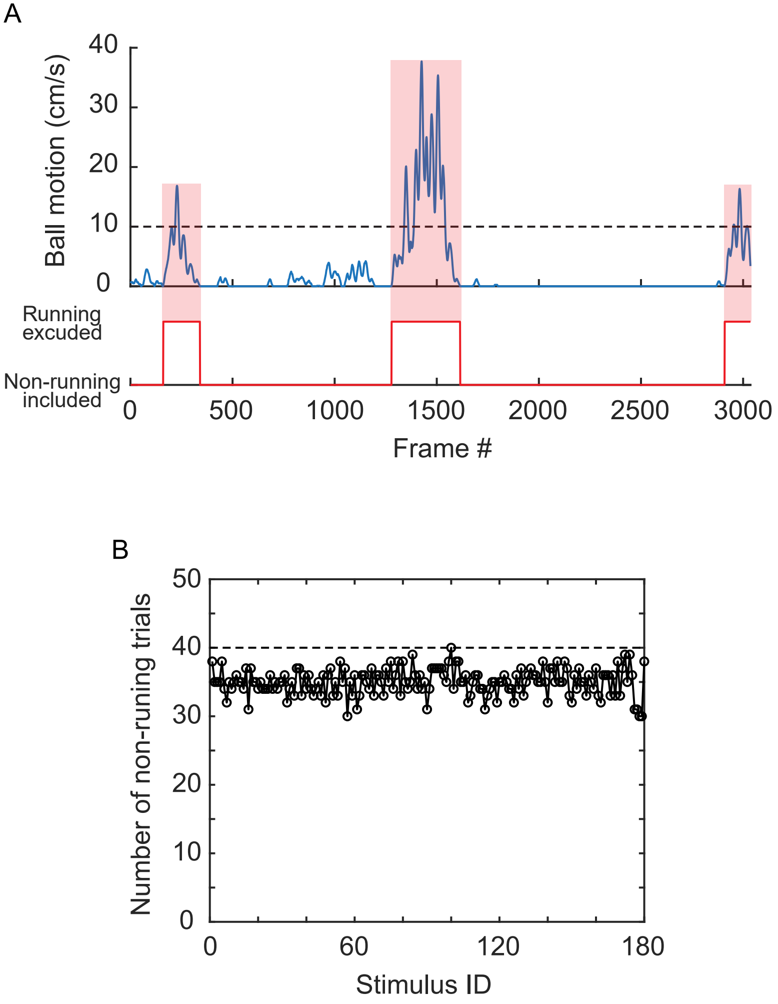

A
Ball motion (cm/s)
40
30
20
10
0
Running
excuded
Non-running
included
0
500
1000
1500
2000
2500
3000
Frame #
B
Number of non-runing trials
50
40
30
20
10
0
0
60
120
180
Stimulus ID

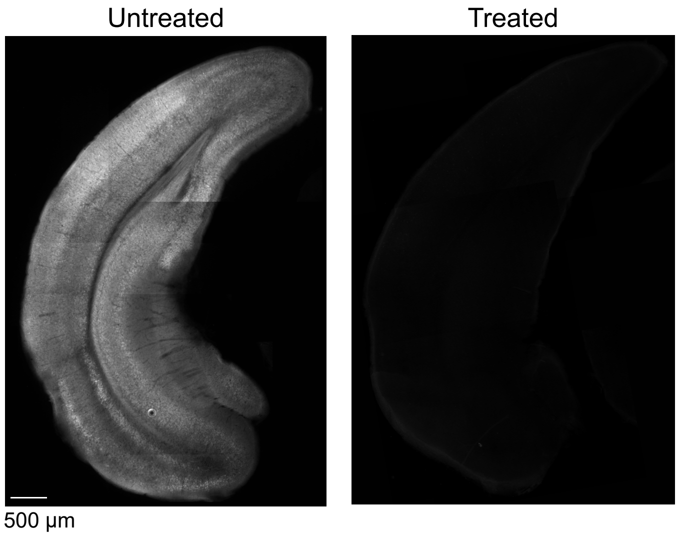
Untreated
Treated
500 μm

Figure S1. Event counts per stimulus bin
Data used to construct 2-D Gaussian plots shown in Figure 1D. Yellow, red, and brown lines correspond to 75%, 50%, 25% of the peak Gaussian fit respectively.

Figure S2. Assessment of deconvolution using reconstructed traces
(A) Trials 1-6, as shown in Figure 1E, were deconvolved. A 6-event threshold was applied and the calcium signal was reconstructed using the temporal kernel shown in 'C'.
(B) Reconstruction in the absence of the 6-event threshold. Arrows indicate the presence of false positive events.
(C) Temporal kernel used in the same imaging session as in 'A' and 'B'.
(D) Distribution of individual neuron values used for the 'noise level' parameter in the same imaging session as A-C.
(E) Distribution of individual neuron values used for the 'neuropil coefficient' parameter in the same imaging session as A-C.

Figure S3. Cross-session registration of successfully tracked neurons
An example of the registration used to identify the same neurons across two sessions. Segments from Session 1 (green) were aligned to segments from Session 2 (pink) by computing a rigid transformation (x-/y-translation and rotation) on the second imaging session with respect to the first imaging session. An individual neuron was considered successfully tracked if the spatial overlap across the two sessions was ≥75%. In this example, 188 neurons were tracked (white overlay). In this example animal, a total of 319 neurons were segmented in Session 1, and 421 neurons were segmented in Session 2.

Figure S4. Signal correlations are highly similar across sessions
Signal correlation matrices for each of the 4 mice (rows) from each session (columns). The similarity of the signal correlation matrices between the two sessions was measured by Pearson's correlation of the upper triangle entries of the signal correlation matrices. All four animals showed a highly similar signal correlation matrices between the two sessions (mouse 1: $r = 0.863$, $p = 1.05e-130$; mouse 2: $r = 0.893$, $p = 0$; mouse 3: $r = 0.902$, $p = 6.31e-57$; mouse 4: $r = 0.772$, $p = 2.60e-305$). The neurons for each animal were sorted by the magnitude of signal correlations in a decreasing order with respect to an arbitrarily chosen neuron from session 1. Days in between imaging Session 1 and Session 2 are indicated for each mouse.

Figure S5. Correlation between orientation preference and bandwidth stability is present when values less than 1 degree are set to zero
(A) Magnitude of change between Session1 and Session2 in preferred orientation versus orientation bandwidth, all values less than 1 degree were set to zero. Spearman's Rank correlation $r=0.328$, $p = 3.38e-5$.
(B) Data re-plotted from Figure 4B, using linear scale for orientation preference values.

Figure S6. Selection of non-running stimulus trials
(A) Identification of running epochs. Ball motion was detected during two behaviors (blue trace), (1) rocking due to apparent weight shifts and (2) running. To identify imaging frames in which

the animal was running, first a threshold of 10 cm/s was applied (dashed line) and second, the duration of the running epoch was defined as any frames contiguous with a threshold-crossing event frame having a ball motion speed greater than zero. Continuous non-zero movement epochs in which the animal exceeded the speed threshold were considered to be running. Frames belonging to such epochs were labelled as running frames (red shading). Visual inspection of the data revealed that ball motion epochs that did not cross the threshold represented periods of rocking due to weight shifts.
(B) Number of non-running trials per stimulus is shown for one complete imaging session for one example mouse. Forty trials of each stimulus were presented (dashed line). Only non-running stimulus trials were used in quantifying a neuron's responsiveness to visual stimulation.

Figure S7. Doxycycline treatment suppresses GCaMP6f expression
Doxycycline included in the food of breeding females is sufficient to suppress GCaMP6f expression. Left, cortical tissue (including V1) from an untreated mouse sacrificed at postnatal day (P) 15. Right, cortical tissue sectioned at a similar anterior-posterior plane, from a P15 mouse treated with doxycycline from conception. Note the faint auto-fluorescent outline of the tissue borders.
